# Effects of Chrysin on Oral Squamous Cell Carcinoma *In Vitro*

**DOI:** 10.1055/s-0042-1755624

**Published:** 2022-09-27

**Authors:** Duangchewan Puengsurin, Supranee Buranapraditkun, Chayanee Leewansangtong, Nitchakarn Taechaaukarakul, Proud Songsivilai, Rudee Surarit, Nakarin Kitkumthorn

**Affiliations:** 1 Department of Oral Biology, Faculty of Dentistry, Mahidol University, Nakhon Pathom, Thailand; 2 Division of Allergy and Clinical Immunology, Department of Medicine, King Chulalongkorn Memorial Hospital, Faculty of Medicine, Chulalongkorn University, Thai Red Cross Society, Bangkok 10330, Thailand; 3 Center of Excellence in Vaccine Research and Development (Chula Vaccine Research Center-Chula VRC), Faculty of Medicine, Chulalongkorn University, Bangkok 10330, Thailand; 4 Thai Pediatric Gastroenterology, Hepatology and Immunology (TPGHAI) Research Unit, King Chulalongkorn Memorial Hospital, Faculty of Medicine, Chulalongkorn University, The Thai Red Cross Society, Bangkok 10330, Thailand

**Keywords:** chrysin, oral squamous cell carcinoma, proliferation, migration, invasion, apoptosis

## Abstract

**Objective** Chrysin is a hydroxylated flavonoid derived from “propolis or bee glue,” a natural product. Previous research on chrysin's biological functions, including anticancer activity, had been reported. However, chrysin's effect on oral squamous cell carcinoma (OSCC) is still scarce. This article aimed to test the cytotoxicity, antiproliferative, antimigration, anti-invasion, and apoptotic effects of purified chrysin in two OSCC cell lines, HSC4 and SCC25.

**Materials and Methods** The malignant phenotype was assessed using cell proliferation, wound healing, and transwell assays. Cell apoptosis was determined using flow cytometry. The positive control was OSCC cells treated with cisplatin, and the negative control was OSCC cells incubated with 0.1% dimethyl sulfoxide.

**Results** Chrysin at concentrations of 100 and 200 µM could inhibit OSCC cell proliferation, migration, and invasion, as well as enhance cell apoptosis, particularly in the early stages of apoptosis.

**Conclusion** In OSCC cell lines, chrysin has been demonstrated to be an effective antioncogenic agent. Additional research is required to confirm the results. Chrysin should be suggested as a possible alternative therapeutic application for OSCC.

## Introduction

Oral squamous cell carcinoma (OSCC) is the most prevalent malignant tumor of the head and neck. These cells, which originated from stratified squamous epithelium, account for 80 to 90% of oral cancer cases. 1 OSCC incidence has gradually increased. 2 Based on estimates from GLOBOCAN 2020 Thailand, 2.5% of all cancer cases and 2% of cancer-related deaths were caused by oral cancer. 2 At present, the mainstream treatment for OSCC is surgical operation combined with radiation therapy and/or chemotherapy, but tumors are still prone to recurrence with a low 5-year survival rate, making patients deformed and suffered from treatment. 3 Therefore, alternative treatments for OSCC are required.

Chrysin is a dihydroxyflavone found in propolis, plants, and mushrooms like passion flowers. 4 Propolis which is a natural resin compound in bee glue has 28 g/L of chrysin, supposedly. 5 Chrysin has two hydroxyl groups at positions 5 and 7 in a flavone's A ring 6 and has antibacterial, antiviral, anti-inflammatory, antioxidant, and anticancer properties. 7 Chrysin has also demonstrated significant potential for inducing apoptosis (programmed cell death) in cancer cells, as well as for inhibiting the proliferation of cancer cells, cancer cell migration, and cancer cell invasion. A study of chrysin's anticancer properties on CAL-27 human tongue carcinoma cells has been reported. 8 The CAL-27 cell line, on the other hand, was identified as an oral adenosquamous carcinoma 9 , a rare variant of OSCC which is not common. 10 For that reason, we focused our research on the effects of chrysin on SCC25 and HSC4, two oral squamous cell lines that have a common histological type. Our objective was to determine the cytotoxic effect of chrysin on OSCC cells *in vitro* , as well as its ability to inhibit OSCC cell proliferation, migration, and invasion.

## Materials and Methods

### Cell Culture

The HSC4 and SCC25 cell lines are non-human papillomavirus OSCC continuous cell lines generated from the human tongue. Both cell lines were obtained from the American Type Culture Collection. HSC4 cells were grown in Dulbecco's Modified Eagle's Medium (DMEM) supplemented with 10% fetal bovine serum (FBS) in a humidified atmosphere of 5% CO _2_ at 37°C. SCC25 cells were grown in a 1:1 mixture of DMEM and Ham's F12 media in a humidified atmosphere of 5% CO _2_ at 37°C.

### Cell Viability Assay

Cell cytotoxicity was measured using the 3-(4,5-dimethylthiazol-2-yl)-2,5-diphenyltetrazolium bromide (MTT) test. 11 12 OSCC cells were seeded at a density of 7 × 10 ^4^ cells per well in a 96-well plate and incubated for 24 hours at 37°C, 5% CO _2_ , and 95% relative humidity. After that, 100 μL of negative control, positive control, and chrysin in six different concentrations (50, 100, 200, 400, 800, and 1000 μM) were added to each well and incubated for another 24 hours. As a negative control, 0.1% dimethyl sulfoxide (DMSO) was used. Each well received 100 μL of MTT solution. After 2 hours of incubation at 37°C, the MTT solution was removed, and each well was washed with 100 μL of phosphate-buffered saline (PBS) solution. Then, 100 μL DMSO was added to each well to dissolve the formazan from the cells. The number of viable cells was related to the amount of formazan produced. The absorbance was measured at 570 nm using an Epoch 2 microtiter spectrophotometer (Agilent Technologies Inc., California, United States). The assay was performed in biological triplicate.

### Cell Proliferation Assay

OSCC cells were seeded in 96-well plates at a density of 2 × 10 ^4^ cells per well and incubated for 24 hours at 37°C, 5% CO _2_ , and 95% relative humidity. The cultured media was then withdrawn. Following that, selected quantities of chrysin (100 and 200 μM) as well as 100 μL of DMSO-dissolved negative and positive controls were added and incubated for 1, 3, 5, and 7 days. After 2 hours of incubation at 37°C in a humidified environment with 5% CO _2_ , in each well, 100 μL of DMSO was added. Finally, using an Epoch 2 microtiter spectrophotometer (Agilent Technologies Inc.), the absorbance was measured at 570 nm. The test was performed in biological triplicate.

### Cell Migration Assay

An *in vitro* wound healing assay was used to assess migration. 13 HSC4 and SCC25 cells were seeded in a 24-well plate with culture medium at a density of 100,000 cells per well. The cells were incubated at 37°C, humidified at 5% CO _2_ environment for 24 hours. The cell monolayer was then scraped off in a straight line with a 1,000 μL pipette tip. After that, 500 μL of 100 and 200 μM chrysin solution were added and incubated for 24 hours. The plate was rinsed with PBS at the end of the incubation period, fixed with ice-cold methanol for 10 minutes, and stained with 0.2% toluidine blue. An inverted microscope was used to photograph cell migration. Finally, the migration zones were determined using the Image-Pro (Media Cybernetics, Maryland, United States) program. The negative control was treated with only media, while the positive control was treated with 100 μM cisplatin. The experiment was done in biological triplicate.

### Cell Invasion Assay

The invasion was assessed using the transwell assay. 14 The transwell insert contains a 24-well cell culture plate with 8 μm pores. The upper transwell chambers were loaded with Matrigel at a density of 100,000 cells per well and were seeded in serum-free DMEM medium. The lower chamber contains 10% FBS in DMEM. The OSCC cells were incubated for 24 hours in 37°C, and humidified at 5% CO _2_ environment after being treated with 100 μL of 100 and 200 μM chrysin. At the end of the incubation period, invaded cells at the bottom of the membrane were fixed and stained. Noninvading cells on the membrane's upper surface were removed by scrubbing with a sterile cotton pellet. The transwell inserts were fixated for 10 minutes in ice-cold ethanol before being stained for 15 minutes with 0.2% crystal violet. Methanol was used to elute the samples, and absorbance was measured and compared with the control. The negative control received only media, while the positive control received 100 μM cisplatin. The test was performed in biological triplicate.

### Apoptosis Assay

Flow cytometry was used to examine the apoptosis of SCC25 and HSC4 cells. Approximately 100,000 cells were stained for 30 minutes at room temperature using Annexin V Alexa Fluor 488 (BioLegend, California, United States) and propidium iodide (PI) (BioLegend). Fluorescence-activated cell sorting buffer was added to the cells for flow cytometry cell type quantification (LSRII, BD Biosciences, California, United States). The cell types and frequencies were then gated using both forward scatter and side scatter. Finally, the data was examined using the FlowJo program (Ashland, Oregon, United States). The test was repeated three times in biological triplicate.

### Statistical Analysis

PASW Statistics 18.0.0 and GraphPad Prism 6.0 were utilized for statistical analysis. To evaluate quantitative data, the Kruskal–Wallis test was performed. The results, which were largely measurements, were shown as mean and standard deviation. A *p* -value of less than 0.05 was considered statistically significant.

## Results

### Cytotoxicity Effect of Chrysin on OSCC Cells

**Fig. 1:**
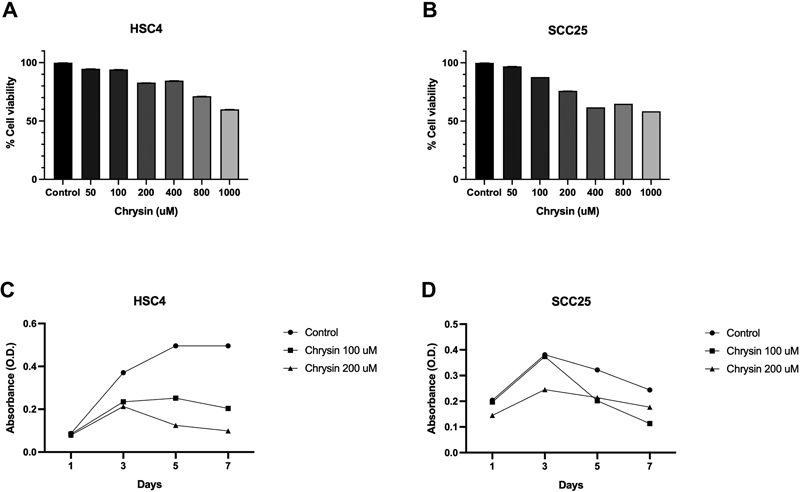
Cytotoxicity effect of chrysin on cell lines in vitro using 3-(4,5-dimethylthiazol-2-yl)-2,5-diphenyltetrazolium bromide (MTT) cell viability assay ( **A** ) HSC4 and ( **B** ) SCC25. The cells were treated with six concentrations of chrysin (50, 100, 200, 400, 800, and 1,000 µM) for 24 hours. The antiproliferative effect of chrysin on cell lines *in vitro* using cell proliferation assay ( **C** ) HSC4, ( **D** ) SCC25. The cells were treated with two concentrations of chrysin (100 and 200 µM) for 1, 3, 5, and 7 days. The negative control contained 0.1% dimethyl sulfoxide (DMSO).

The half-maximal inhibitory concentration (IC50) for any of the chrysin concentrations used in this experiment (50, 100, 200, 400, 800, and 1,000 µM) for both HSC4 and SCC25 was not measured. Chrysin-treated HSC4 cells showed a gradual decrease in cell viability from 50 to 1,000 µM concentration of chrysin ( Fig. 1A ). Similarly, from 50 to 1000 µM concentration of chrysin, the percent cell viability of chrysin-treated SCC25 cells steadily declined ( Fig. 1B ). At 100 and 200 µM, chrysin showed that more than 75% of the cells in both cell lines were still alive. So, 100 and 200 µM of chrysin were chosen as the best amounts for further tests. Supplementary Table S1 (available in the online version) provides more information on the data shown.

### Antiproliferative Effect of Chrysin on OSCC Cells

HSC4 cells treated with 100 µM concentration of chrysin showed a gradual decrease in absorbance after day 5 after of incubation. But after day 3, 200 µM chrysin drastically decreased the absorbance of HSC4 cells ( Fig. 1C ). For the SCC25 cell line, 100 and 200 µM chrysin-treated cells exhibited gradual decreases in absorbance after day 3 with statistical significance when compared with the control group ( Fig. 1D ). As a consequence, chrysin at 100 and 200 µM were suggested to have the ability to inhibit proliferation. Additional details about this data can be found in Supplementary Table S2 .

### Antimigratory and Anti-Invasion Effect of Chrysin on OSCC Cells

**Fig. 2:**
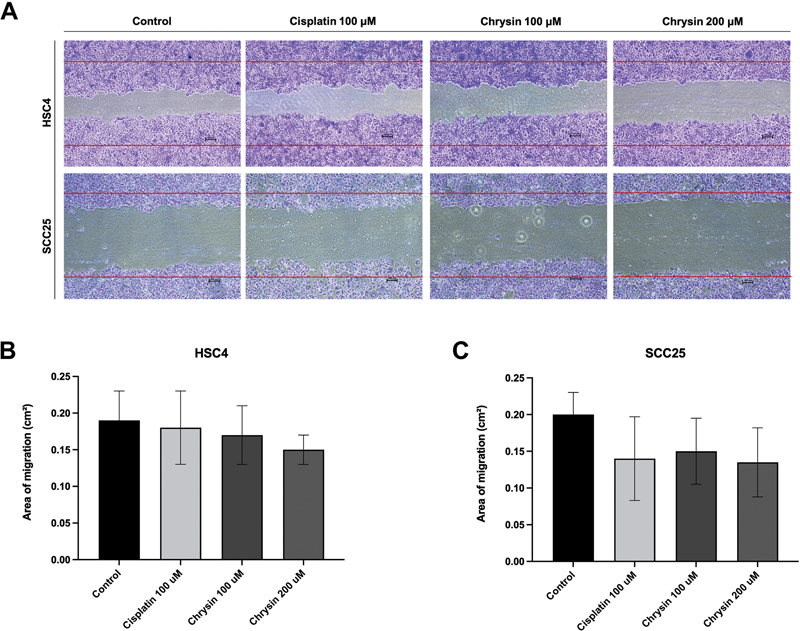
Antimigratory effect of chrysin on oral squamous cell carcinoma (OSCC) cell lines *in vitro* using wound-healing assay. HSC4 and SCC25 cells were treated with two concentrations of chrysin (100 and 200 µM) for 24 hours, compared with the negative control (0.1% dimethyl sulfoxide [DMSO]) and the positive control (100 µM cisplatin). ( **A** ) Microscopic pictures. ( **B** ) Histogram of HSC4. ( **C** ) Histogram of SCC25.

In comparison to the control group, HSC4 and SCC25 treated with chrysin 100 and 200 µM showed a concentration-dependent reduction in migratory regions ( Fig. 2A–C , Supplementary Table S3 , available in the online version). The ability to reduce cell migration is comparable to that of cisplatin-treated cells.

**Fig. 3:**
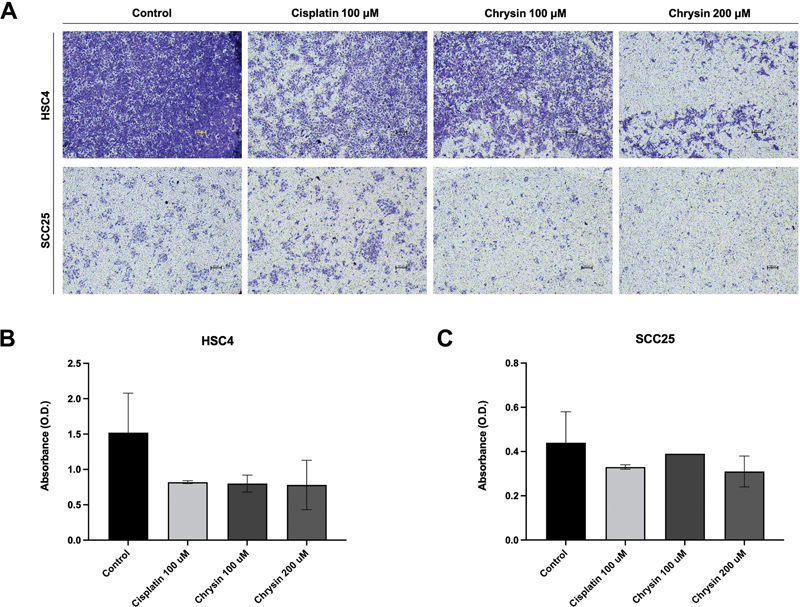
Anti-invasion effect of chrysin on oral squamous cell carcinoma (OSCC) cell lines *in vitro* using transwell assay. HSC4 and SCC25 cells were treated with two concentrations of chrysin (100 and 200 µM) for 24 hours, compared with the negative control (0.1% dimethyl sulfoxide [DMSO]) and the positive control (100 µM cisplatin). ( **A** ) Microscopic pictures. ( **B** ) Histogram of HSC4. ( **C** ) Histogram of SCC25.

Chrysin-treated HSC4 and SCC25 showed a concentration-dependent reduction in absorbance and percentage of invasion as compared with the control group ( Fig. 3A–C , Supplementary Table S4 ). Similar to the migratory effect, the ability to reduce cell invasion is equivalent to that of cisplatin-treated cells

### Chrysin Enhance Apoptotic Effect of OSCC Cells

**Fig. 4:**
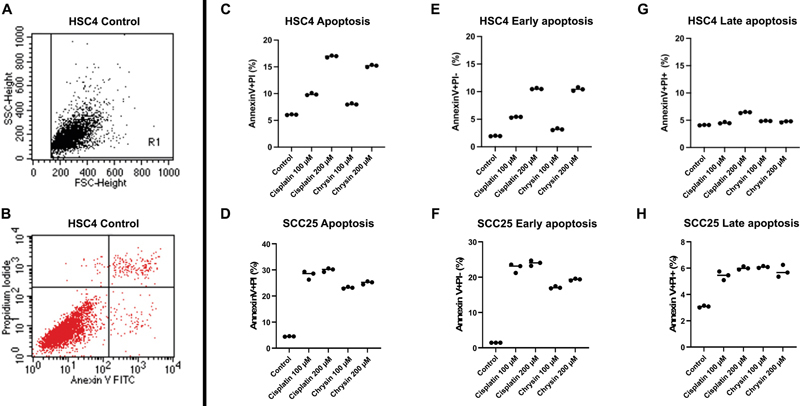
Apoptotic effect of chrysin on oral squamous cell carcinoma (OSCC) cell lines *in vitro* using flow cytometry assay. HSC4 and SCC25 cells were stained with annexin V and propidium iodide (PI). Both cells treated with two concentrations of chrysin (100 and 200 µM) for 24 hours, compared with the negative control (0.1% dimethyl sulfoxide [DMSO]) and the positive control (100 µM cisplatin). ( **A** , **B** ) Flow cytometry gating. Histogram of ( **C** , **D** ) total apoptosis, ( **E** , **F** ) early apoptosis, and ( **G** , **H** ) late apoptosis.

The flow cytometry results were gated and exemplified in Fig. 4A and B . According to annexin-V/PI staining, SCC25 and HSC4 cells treated with chrysin had a higher rate of apoptosis than the control and a similar level to cisplatin-treated cells. The apoptotic rate of HSC4 cells is influenced by chrysin concentration, especially early apoptosis. In comparison to 100 μM chrysin-treated OSCC cells, 200 μM chrysin-treated OSCC cells have a higher percentage of early apoptosis and a similar percentage of late apoptosis, indicating that chrysin has a significant impact on early apoptosis ( Fig. 4C–H ). Additional information on the apoptosis data is available in Supplementary Table S5 , available in the online version.

## Discussion

Our study aims to explore chrysin's antiproliferative, antimigratory, anti-invasion, and apoptotic effects on HSC4 and SCC25. The two cell lines, HSC4 and SCC25, are both OSCC cell lines that were originally excised from the human tongue. However, chrysin has been shown to affect the two cell lines differently. This is possibly due to the different characteristics of the two cell lines. HSC4 is a metastasized cancer from the cervical lymph node. 15 Meanwhile, SCC25 is a primary OSCC of the tongue. 16 Resistance to cytotoxic agents is common in metastatic tumors, which corresponds to our findings.

In the present study, the cytotoxic potential of chrysin on HSC4 and SCC25 cell lines was evaluated using the MTT cell viability assay. As a result, the viability of chrysin-treated OSCC cells gradually decreased as the concentration of chrysin was increased. However, cell viability of HSC4 cells treated with a 400 µM concentration of chrysin, as well as SCC25 cells treated with 800 µM chrysin, slightly increased compared with previous concentrations. Even though the experiment was repeated three times, errors could have occurred due to interexperiment and intraexperiment variations. Furthermore, because all concentrations of chrysin in this experiment were not assessed with the half-maximal inhibitory concentration, chrysin under 1,000 μM showed no significant inhibitory effect against both HSC4 and SCC25 cell lines from the IC50. Chrysin at 100 and 200 µM concentrations increased cell viability in both OSCC cell lines by more than 75%. Therefore, 100 and 200 µM concentrations of chrysin were selected for further experiments due to their low toxicity. Chrysin has been reported to suppress proliferative activity in various cancer cells. 6 17 Likewise, the findings of this study revealed that chrysin has antiproliferative effects against HSC4 and SCC25 cell lines at 100 and 200 µM concentrations, the latter of which is superior.

OSCC has a highly invasive nature along with a high rate of lymph node metastasis. Metastasis of cancer is a complex process in which the primary tumor disseminates and travels to distant organs, resulting in the growing tumor within secondary sites. 18 Cancer cells can spread by several cellular processes, such as cell migration and cell invasion. 19

Cell migration refers to the ability of cells to move within the extracellular matrix, and the process is recreated in vitro as the wound healing assay. 13 18 At 100 and 200 µM concentration, chrysin exhibited a significant antimigratory effect on OSCC cell lines (HSC4 and SCC25), resulting in a decrease of migrated areas compared with control within a 24-hour time interval. Moreover, the result shows that 200 µM chrysin antimigration effects on both HSC4 and SCC25 cell lines are superior to the positive control (100 µM cisplatin). Cell invasion refers to the ability of cells to invade through the basement membrane. The process is recreated in vitro by the transwell assay. The results have shown that at 100 and 200 µM concentrations, chrysin has effectively inhibited the cell invasion of both OSCC cell lines (HSC4 and SCC25). Furthermore, the results revealed that the anti-invasion effects of 200 µM chrysin on both HSC4 and SCC25 cell lines are comparable to the positive control (100 µM cisplatin). Together, chrysin may be better or comparable to cisplatin in its ability to inhibit OSCC invasion and metastasis.

Several studies in different cancer cell lines found that chrysin increased apoptosis. 20 21 22 23 24 Furthermore, chrysin was discovered to significantly induce both early and late apoptosis in cervical cancer cells 25 and prostate cancer cell lines. 26 According to our findings, chrysin has a greater impact on early apoptosis. These findings support the idea that chrysin primarily affects tumor growth by inducing apoptosis via death receptors and/or activating proapoptotic members of the B cell lymphoma 2 (Bcl-2) pathway. 27 28

The limitations of the study were that all experiments were conducted *in vitro* and on only two cell lines (HSC4 and SCC25). To determine the clinical efficacy of chrysin on OSCC cells, additional *in vitro* and *in vivo* studies on additional OSCC cell lines are required. In addition, the precise mechanism underlying chrysin's antimigratory and anti-invasion effects on OSCC cells should be investigated in greater detail.

## Conclusion

The current study collectively characterized the critical role of chrysin on OSCC cells, as it could inhibit their proliferation, migration, and invasion abilities, while enhancing cell apoptosis. And hence, 100 and 200 µM chrysin possessed the potential to reduce the metastatic ability of OSCC. In particular, 200 μM chrysin has equivalent antimigratory and anti-invasion effects compared with 100 μM cisplatin. And 200 µM of chrysin is better compared with 100 µM of chrysin for antimigratory, anti-invasion, and antiproliferative applications. As a result, chrysin 200 μM can be used in conjunction with current OSCC treatment. Our opinion is that chrysin may be suggested for topical application in selected circumstances.
