# Supplementary material for: Effects of Chrysin on Oral Squamous Cell Carcinoma In Vitro
Source: Eur J Dent. 2022 Sep 27;17(3):797–803. doi: 10.1055/s-0042-1755624 (PMC10569856; doi:10.1055/s-0042-1755624)
Supplement: Supplementary file 1 — Supplementary Material [file 10-1055-s-0042-1755624-s2262150.pdf]

Supplementary Table S1 Cytotoxicity effect of chrysin on OSCC cell lines *in vitro* using MTT cell viability assay

Cell line	Compounds	Concentration (μM)	Cell viability (%)	Standard deviation (SD, %)
HSC4	Control	–	100	0.053
	Chrysin	50	94.75	0.069
		100	94.09	0.07
		200	82.87	0.034
		400	84.56	0.05
		800	71.29	0.03
		1,000	60.03	0.047
SCC25	Control	–	100	0.017
	Chrysin	50	96.98	0.016
		100	87.75	0.014
		200	75.9	0.012
		400	61.84	0.014
		800	64.85	0.021
		1,000	58.43	0.013

Abbreviations: MTT, 3-(4,5-dimethylthiazol-2-yl)-2,5-diphenyltetrazolium bromide; OSCC, oral squamous cell carcinoma.

Supplementary Table S2 Antiproliferative effect of chrysin on OSCC cell lines *in vitro* using cell proliferation assay

Cell line	Compound	Chrysin concentration (μM)	Time(d)	Absorbance(OD)	Standard deviation(SD)
HSC4	Control	–	1	0.087	0.008
			3	0.371	0.057
			5	0.496	0.094
			7	0.496	0.128
	Chrysin	100	1	0.084	0.005
			3	0.235	0.014
			5	0.252	0.033
			7	0.204	0.038
		200	1	0.079	0.005
			3	0.213	0.041
			5	0.125	0.021
			7	0.099	0.011
SCC25	Control	–	1	0.204	0.004
			3	0.381	0.019
			5	0.322	0.017
			7	0.244	0.045
	Chrysin	100	1	0.197	0.01
			3	0.374	0.017
			5	0.202	0.025
			7	0.113	0.032
		200	1	0.145	0.021
			3	0.245	0.013
			5	0.214	0.014
			7	0.177	0.025

Abbreviations: OD, optical density; OSCC, oral squamous cell carcinoma.

 © 2022. The Author(s).

Supplementary Table S3 Antimigratory effect of chrysin on OSCC cell lines *in vitro* using wound-healing assay

Cell line	Compound	Concentration (μM)	Area of migration (cm^2)	Standard deviation (SD, cm^2)
HSC4	Control	–	0.191	0.041
	Chrysin	100	0.174	0.038
		200	0.154	0.022
	Cisplatin	100	0.185	0.053
SCC25	Control	–	0.199	0.029
	Chrysin	100	0.152	0.045
		200	0.135	0.047
	Cisplatin	100	0.138	0.057

Abbreviation: OSCC, oral squamous cell carcinoma.

Supplementary Table S4 Anti-invasion effect of chrysin on OSCC cell lines *in vitro* using transwell assay

Cell line	Compound	Concentration (μM)	Absorbance(OD)	Standard deviation(SD)
HSC4	Control	–	1.52	0.557
	Chrysin	100	0.799	0.117
		200	0.776	0.352
	Cisplatin	100	0.821	0.023
SCC25	Control	–	0.438	0.14
	Chrysin	100	0.388	0.002
		200	0.388	0.068
	Cisplatin	100	0.327	0.014

Abbreviations: OD, optical density; OSCC, oral squamous cell carcinoma.

Supplementary Table S5 Apoptotic effect of chrysin on OSCC cell lines *in vitro* using flow cytometry

Cell line	Compounds	Concentration (μM)	Necrosis (%)	Early apoptosis (%)	Late apoptosis (%)	Total apoptosis (%)	Viable cells (%)
HSC4	Control	–	0.66	1.95	4.10	6.05	93.28
	Chrysin	100	0.53	3.17	4.87	8.04	91.43
		200	0.61	10.46	4.73	15.19	84.21
	Cisplatin	100	0.95	5.37	4.52	9.89	89.16
SCC25	Control	–	7.28	1.44	3.08	4.52	88.20
	Chrysin	100	1.11	17.09	6.09	23.17	75.71
		200	0.90	19.34	5.75	25.10	74.01
	Cisplatin	100	0.85	22.62	5.43	28.05	71.10

Abbreviation: OSCC, oral squamous cell carcinoma.

 © 2022. The Author(s).
